# Construction of a Compound Model to Enhance the Accuracy of Hepatic Fat Fraction Estimation with Quantitative Ultrasound

**DOI:** 10.3390/diagnostics15020203

**Published:** 2025-01-17

**Authors:** Zsély Boglárka, Zita Zsombor, Aladár D. Rónaszéki, Anna Egresi, Róbert Stollmayer, Marco Himsel, Viktor Bérczi, Ildikó Kalina, Klára Werling, Gabriella Győri, Pál Maurovich-Horvat, Anikó Folhoffer, Krisztina Hagymási, Pál Novák Kaposi

**Affiliations:** 1 Department of Radiology, Medical Imaging Centre, Semmelweis University, 1082 Budapest, Hungary; zsely.boglarka@stud.semmelweis.hu (Z.B.); zsombor.zita@stud.semmelweis.hu (Z.Z.); ronaszeki.aladar.david@semmelweis.hu (A.D.R.); robert.stollmayer@med.uni-heidelberg.de (R.S.); marco.himsel@stud.semmelweis.hu (M.H.); berczi.viktor@semmelweis.hu (V.B.); kalina.ildiko@semmelweis.hu (I.K.); gyori.gabriella@semmelweis.hu (G.G.); maurovich-horvat.pal@semmelweis.hu (P.M.-H.); 2 Department of Surgery, Transplantation, and Gastroenterology, Semmelweis University, 1082 Budapest, Hungary; egresi.anna@semmelweis.hu (A.E.); werling.klara@med.semmelweis.hu (K.W.); hagymasi.krisztina@med.semmelweis.hu (K.H.); 3 Clinic for Diagnostic and Interventional Radiology (DIR), Heidelberg University Hospital, 69120 Heidelberg, Germany; 4 Department of Internal Medicine and Oncology, Semmelweis University, 1082 Budapest, Hungary; folhoffer.aniko@semmelweis.hu

**Keywords:** fatty liver, metabolic dysfunction-associated steatotic liver disease, ultrasound fat fraction (USFF), magnetic resonance imaging proton-density fat fraction, regression analysis

## Abstract

**Background:** we evaluated regression models based on quantitative ultrasound (QUS) parameters and compared them with a vendor-provided method for calculating the ultrasound fat fraction (USFF) in metabolic dysfunction-associated steatotic liver disease (MASLD). **Methods:** We measured the attenuation coefficient (AC) and the backscatter-distribution coefficient (BSC-D) and determined the USFF during a liver ultrasound and calculated the magnetic resonance imaging proton-density fat fraction (MRI-PDFF) and steatosis grade (S0–S4) in a combined retrospective–prospective cohort. We trained multiple models using single or various QUS parameters as independent variables to forecast MRI-PDFF. Linear and nonlinear models were trained during five-time repeated three-fold cross-validation in a retrospectively collected dataset of 60 MASLD cases. We calculated the models’ Pearson correlation (r) and the intraclass correlation coefficient (ICC) in a prospectively collected test set of 57 MASLD cases. **Results:** The linear multivariable model (r = 0.602, ICC = 0.529) and USFF (r = 0.576, ICC = 0.54) were more reliable in S0- and S1-grade steatosis than the nonlinear multivariable model (r = 0.492, ICC = 0.461). In S2 and S3 grades, the nonlinear multivariable (r = 0.377, ICC = 0.32) and AC-only (r = 0.375, ICC = 0.313) models’ approximated correlation and agreement surpassed that of the multivariable linear model (r = 0.394, ICC = 0.265). We searched a QUS parameter grid to find the optimal thresholds (AC ≥ 0.84 dB/cm/MHz, BSC-D ≥ 105), above which switching from a linear (r = 0.752, ICC = 0.715) to a nonlinear multivariable (r = 0.719, ICC = 0.641) model could improve the overall fit (r = 0.775, ICC = 0.718). **Conclusions:** The USFF and linear multivariable models are robust in diagnosing low-grade steatosis. Switching to a nonlinear model could enhance the fit to MRI-PDFF in advanced steatosis.

## 1. Introduction

Steatotic liver disease is one of the most common chronic liver conditions, and it represents a major challenge to healthcare systems worldwide [1]. An international consensus statement recommended a recent change in the nomenclature of liver steatosis which introduced positive diagnostic criteria for diagnosing metabolic dysfunction-associated steatotic liver disease (MASLD) [2]. MASLD is defined by fat vacuoles in more than 5% of the hepatocytes and at least one of five cardiometabolic risk factors. The cases with evidence of active hepatitis are classified as metabolic dysfunction-associated steatohepatitis (MASH). The worldwide prevalence of MASLD is 38% among adults and 15–20% among children, and it is expected to grow during the next decade [3]. MASLD is linked to an increased prevalence of type 2 diabetes (T2DM), higher risk of mortality, cardiovascular disease, hepatocellular carcinoma, and non-hepatic cancers [3]. MASH is also the leading cause of liver transplantation among women and the most rapidly growing indication of all transplantations [4].

There is a growing demand for accessible, noninvasive methods capable of accurate, objective assessment of liver steatosis, streamlining the diagnosis of MASLD. In inflammatory bowel disease, ultrasound screening can facilitate the detection of associated metabolic dysregulation and early detection of MASLD [5]. Conventionally, liver biopsy was the gold standard diagnostic method of steatosis. However, its diagnostic use is limited by low accessibility, high cost, interrater variability, and non-negligible risk of complication [6]. Liver biopsy is mainly reserved for demonstrating active steatohepatitis in MASH and when there is a disagreement between noninvasive tests [7]. Multiple clinical and laboratory scoring systems have been proposed for biopsy-free diagnosis of liver steatosis in MASLD [8]. In general, clinical scores have lower sensitivity for low-grade steatosis than imaging studies, and some require measurement of specific serum markers, which are not universally available in routine practice [9]. Magnetic resonance H^1^ spectroscopy (MRS) and chemical-shift-encoded magnetic resonance imaging (CSE-MRI) proton-density fat fraction (MRI-PDFF) are currently the most sensitive and accurate noninvasive methods for the detection of liver steatosis [10,11]. MRI-PDFF can be calculated for the entire liver volume, allowing for precise quantification even when the fat is unevenly distributed. Meanwhile, these methods are unsuitable for screening large patient populations due to the high cost and limited availability of MRI scans.

Ultrasound has many advantages over other imaging modalities, such as low cost, excellent availability, avoidance of X-ray radiation, and real-time anatomy visualization from multiple planes. Steatosis causes well-recognizable changes in the ultrasound appearance of the liver, including increased reflectivity and posterior attenuation, but these can only be reliably detected at >20% fat fraction [12]. Physical parameters assessed by quantitative ultrasound (QUS), such as the controlled attenuation parameter (CAP), attenuation coefficient (AC), backscatter coefficient (BSC), and backscatter-distribution coefficient (BSC-D), have improved sensitivity and reproducibility compared to B-mode scans and allow for reliable detection of ≥5% liver steatosis [13,14,15]. The impediments of QUS are that diagnostic thresholds can differ between instruments, the interpretation of physical quantities can be challenging in a clinical setting, and parameter values plateau in high-grade steatosis. Various mathematical models have recently been advised to calculate an ultrasound fat fraction (USFF) as a percentage, similar to MRI-PDFF, based on a combination of QUS parameters. Some models use linear or nonlinear regression analysis, while others utilize deep learning analysis to estimate USFF [16,17,18,19]. Our research group has also developed and validated a nonlinear least-squares (NLLS) model built on QUS parameters for the calculation of an ultrasound-estimated fat fraction (UEFF) [20].

Only a handful of reports have attempted to compare fat fraction models. In the present study, we evaluated and compared multiple regression-based methods side by side. We also advise a compound prediction strategy for optimizing estimates in all grades of steatosis.

## 2. Materials and Methods

The Regional and Institutional Science and Research Ethics Committee of our university has approved the study protocol. Subject enrollment, scans, data collection, and processing all comply with the World Medical Association Declaration of Helsinki, revised in Edinburgh in 2000, and our institutional standards. The subjects have given written informed consent to participate in this combined retrospective–prospective cohort study.

### 2.1. Patient Selection

In our institution, we screened four hundred seventy-two subjects for liver steatosis with QUS between July 2020 and February 2024, whose data were retrospectively reviewed for this research. We prospectively enrolled fifty-seven patients whose clinical findings were consistent with MASLD and were referred for a QUS scan to confirm the diagnosis between April 2024 and October 2024 in our institution.

The inclusion criteria were the following: age of 18 years or older; clinical presentation was consistent with MASLD or MASH and included at least one metabolic risk factor as described in the multisociety consensus statement [2]; self-reported daily alcohol consumption was less than 30 g (3 drinks) in the case of men or 20 g (2 drinks) in the case of women in the past two years; patients who had both QUS and CSE-MRI in our institution; those who gave written informed consent to participate in this study. The exclusion criteria were the following: age of less than 18 years, pregnancy, history of known chronic liver disease other than MASLD or MASH, decompensated chronic liver disease, a significant amount of ascites, acute cholestasis, acute or acute on chronic liver failure, secondary liver steatosis due to exposure to hepatotoxic agents and other specific etiologies (i.e., hemochromatosis), or large focal lesions hindering QUS.

The retrospectively collected cases were reviewed for inclusion and exclusion criteria, and a group of sixty eligible subjects was selected with approximately equal representation of steatosis grades (12 subjects with S2 grade, as well as 16 subjects each with S0, S1, and S3 grades). All prospectively collected cases were enrolled. Thus, the final study cohort contained 117 subjects.

### 2.2. Quantitative Ultrasound and USFF Measurements

The QUS and USFF were measured during a regular outpatient visit by one of five expert physicians with at least five years of experience in liver ultrasound in our institution. All scans were performed with a Samsung RS85 Prestige system (Samsung Medison Co., Ltd., Seoul, Republic of Korea) using the CA 1-7S convex probe (Samsung Medison Co., Ltd., Seoul, Republic of Korea). Before March 2024, we could measure the tissue attenuation imaging (TAI^TM^) coefficient, an equivalent to the AC, and the tissue scatter-distribution (TSI^TM^) coefficient, an equivalent to BSC-D. After a software update in March 2024, the percentage of USFF was also calculated by the QUS application from five TAI and TSI data points [17]. The unit of TAI was dB/cm/MHz. The TSI had no physical unit.

**Figure 1:**
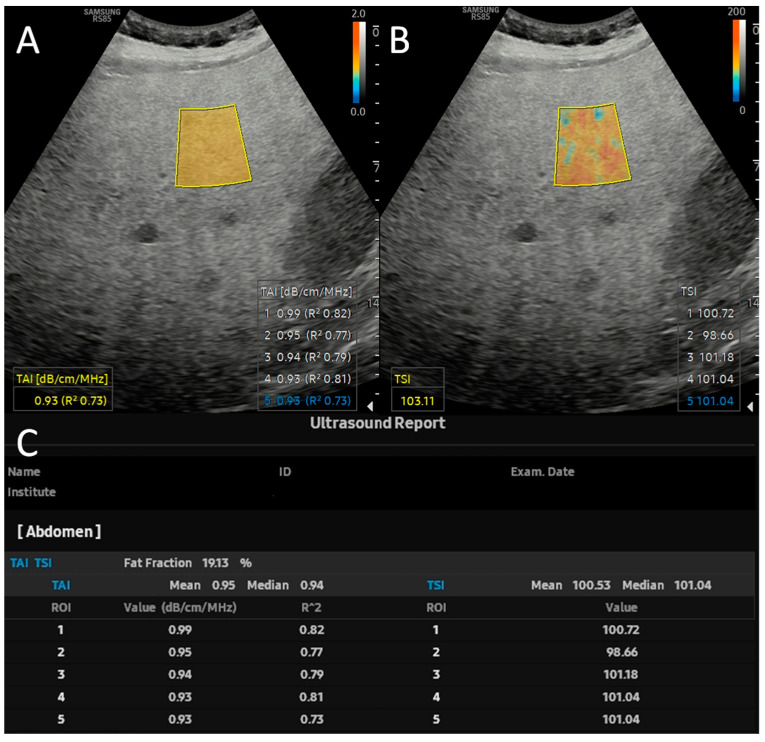
Quantitative ultrasound measurement and calculation of ultrasound fat fraction. (**A**) The tissue attenuation coefficient (TAI^TM^), equivalent to the attenuation coefficient (AC), and (**B**) the tissue scatter-distribution coefficient (TSI^TM^), equivalent to the backscatter-distribution coefficient (BSC-D), are measured five times inside a color-coded window placed by the examiner in the right lobe 2 cm from the liver capsule. (**C**) The software automatically calculated the ultrasound fat fraction (USFF) from five TAI and TSI values and displayed it in the quantitative report.

Patients were asked to fast for four hours before the scan. During QUS, the patients were supine with a right hand elevated above the head. The right lobe of the liver was visualized through an intercostal space during a shallow breath hold while the probe was held perpendicular to the skin surface. The examiner placed the color-coded fan-shaped measurement window in the liver 2 cm below the capsule. The TAI values with an R-squared (R^2^) < 0.7 were considered non-reliable and were discarded. TAI and TSI were measured five times; the averages and, when available, the USFF were recorded (Figure 1). The skin-to-liver distance (SLD) was measured manually using B-mode ultrasound images.

We also measured the liver stiffness (LS) using two-dimensional shear wave elastography (2D-SWE, S-Shearwave Imaging^TM^, Samsung Medison Co., Ltd., Seoul, Republic of Korea). The scanning protocol for obtaining LS was identical to that of QUS. The LS was measured in kPa units. The LS values with a reliable measure index < 0.4, indicative of a low signal-to-noise ratio, were discarded. The measurements were repeated until at least five valid LS values could be gathered, and the average and the interquartile range/median (IQR/M) ratio were recorded. If the IQR/M was ≥30%, the measurement was considered non-reproducible and repeated. The cases were classified into four fibrosis grades (F0/1, F2, F3, and F4) using cut-off values of 5 kPa, 9 kPa, and 13 kPa [21].

### 2.3. Chemical-Shift-Encoded Quantitative MRI

We performed CSE-MRI to measure MRI-PDFF, which was used as a reference standard of liver fat quantification. All study subjects underwent a CSE-MRI preferably within a week but no longer than one month of the QUS. The patients were scanned in a Philips Ingenia^TM^ 1.5T scanner (Philips Healthcare, Amsterdam, Netherlands) using a multichannel Q-body^TM^ coil (Philips Healthcare, Amsterdam, the Netherlands). A detailed description of the scanning parameters has been published previously and is provided in the Supplementary Materials (Supplementary Table S1) [11]. The quantitative fat fraction maps were reconstructed with multi-scale quadratic pseudo-Boolean optimization with graph-cut algorithm (https://github.com/bretglun/fwqpbo, accessed on 20 July 2023) with multipeak fat spectrum modeling in a Python 3.9.7 environment (https://python.org, accessed on 20 July 2023) [22]. The MRI-PDFF was measured as the average of three identical approximately 5 cm^2^ circular regions of interest (ROIs) placed by an expert radiologist in the right lobe of the liver at the level of porta hepatis using the ImageJ 1.53e software (https://imagej.net/ij, accessed on 20 July 2023). The cases were classified into steatosis grades (S0–S3) based on MRI-PDFF with cut-off values of 5%, 15%, and 20% [17].

### 2.4. Training and Validation of QUS-Based Prediction Models

We split the study population into similar-size training and test sets. The retrospectively collected dataset consisted of sixty cases with a balanced representation of steatosis grades (S0: 16 cases, S1: 16 cases, S2: 12 cases, and S3: 16 cases). Meanwhile, the fifty-seven prospectively enrolled cases for whom USFF was also available were assigned to the test set.

We constructed five types of nonlinear and five linear models using different sets of predictor variables to forecast MRI-PDFF. The linear models were built using the standard regression formula MRI-PDFF = beta 1 × X*_n_* + beta 2, where X*_n_* is the value of the *n*th predictor variable, and beta (b)1 and b2 are the model’s coefficients. The nonlinear models were built with NLLS analysis where MRI-PDFF = b1 × exp(b2 × X*_n_*) + b3, and b1, b2, and b3 are the model’s coefficients. The simple models used either AC or BSC-D as the predictor variable. Meanwhile, the predictors in the multivariable models were both AC and BSC-D. We also tested the effects of body mass index (BMI) and SLD by adding them to the multivariable models as a third independent variable.

The optimal model parameters were determined using five-times-repeated three-fold cross-validation in the training set. Finally, we validated and compared the accuracy of the best-fit models in the test set.

### 2.5. Statistical Analysis

We report the continuous variables as mean ± standard deviation (SD) and categorical variables as number (*n*) and percentage (%). We performed a Fisher exact test to evaluate the odds ratios (ORs) of steatosis grades in the test set. Multivariable and multiple simple regression models were built to select ultrasound parameters and clinical variables that significantly correlate with MRI-PDFF as the outcome variable. We reported the F-statistics (F), the first-order coefficient (b), and the significance level (*p*) to describe the relationship between outcome and predictor variables. We calculated the natural logarithm (log) of the likelihood function (LL) for the trained models. The log-likelihood ratios (LRs) with significance were used to decide if the models differed in the goodness of fit. The Pearson correlation coefficient (r) and the squared correlation coefficient (R^2^) were calculated to assess the strength of the linear relationship between the models’ predictions and MRI-PDFF in the test set. We also calculated the intraclass correlation coefficient (ICC) for absolute agreement using single units in a two-way model to quantify the agreement between MRI-PDFF and predicted fat fractions. We constructed Bland–Altman plots to visualize the mean bias and the limits of agreement (LOAs) between compound predictions and USFF.

The *p* < 0.05 was used as the threshold of statistical significance. The Bonferroni method was used to control the Type I error rate in multiple comparisons. The training of the prediction models and statistical analysis were performed in the R version 4.3.3 (www.r-project.org, accessed on 5 March 2024) environment.

## 3. Results

### 3.1. Demographics and Clinical Characteristics of the Study Cohort

We split the study population into similarly sized training (*n* = 60) and test (*n* = 57) sets. The patients were retrospectively assigned to the training set to achieve a balanced representation of all grades, including 16/60 (26.7%) S0, S1, and S3 and 12/60 (20%) cases of S2-grade steatosis. Meanwhile, the test set was collected prospectively, including all patients screened for liver steatosis with USFF. Thus, the proportion of cases in the test set with no steatosis (3/57, 5.3%) was significantly lower than with S1-grade (23/57, 40.4%, OR = 0.084, 95% CI: 0.015–0.3091, *p* < 0.001), S2-grade (12/57, 21.1%, OR = 0.211, 95% CI: 0.036–0.848, *p* = 0.024), and S3-grade (19/57, 33.3%, OR = 0.113, 95% CI: 0.020–0.424, *p* < 0.001) steatosis. The details of demographics and clinical data of the training and test patient cohorts are summarized in Table 1.

The proportions of females (26/60, 43.3%) and males (34/60, 56.6%) were almost equal in the training set. Patients were typically overweight, with a mean BMI ± SD of 29.8 ± 4.18 kg/m^2^, and a quarter of them (15/60, 25%) were also diagnosed with T2DM. Most of the patients in the training set did not have significant liver fibrosis (F3 or F4 grade) based on the assessment with 2D-SWE (F0/1 grade: 7/60, 11.6%; F2 grade: 40/60, 66.6%; F3 grade: 9/60, 15%; F4 grade: 4/60, 6.6%).

The test set also included a nearly equal number of females (26/57, 45.6%) and males (31/57, 54.4%). The BMI (30.57 ± 13.16 kg/m^2^) was similarly high in the test as in the training group, but T2DM (7/57, 12.3%) was less frequent. The majority of the test cases had non-significant fibrosis (F0/1 grade: 35/57, 61.4%; F2 grade: 17/57, 29.8%; F3 grade: 3/5.3%; F4 grade: 2/57, 3.5%).

### 3.2. Variable Selection and Training of Models

We performed linear regression analyses on the training set to identify basic clinical and ultrasound parameters that can be used to forecast MRI-PDFF (Table 2). The AC was the only independent predictor (F = 8.39, b = 47.7, adj. R^2^ = 0.494, *p* < 0.001) of MRI-PDFF in the multivariable test. In the univariable analysis, AC (F = 66.28, b = 53.04, R^2^ = 0.533, *p* < 0.001) and BSC-D (F = 20.96, b = 0.573, R^2^ = 0.265, *p* < 0.001) showed significant association with MRI-PDFF, while age, BMI, gender, LS, and SLD did not.

We constructed multiple linear and nonlinear models using a single ultrasound parameter or a combination of two ultrasound parameters and trained them using five-times-repeated three-fold cross-validation. The log-likelihood test indicated that the fit of simple AC models was similar to the combined AC and BSC-D models (linear models: LL = −182.01 vs. LL = −181.48, *p* = 0.305; nonlinear models: LL = −181.51 vs. LL: −181.51, *p* = 0.999). The fit of simple BSC-D models was significantly worse (linear LL: −195.13, *p* < 0.001, nonlinear LL: −194.86, *p* < 0.001). The comparisons of the corresponding linear and nonlinear models did not reveal a significant difference (*p* = 0.999). Adding either BMI (linear LL: −181.47, nonlinear LL: −194.37) or SLD (linear LL: −181.45, nonlinear LL: −181.68) to the multivariable models as a third predictor variable did not improve the fit.

### 3.3. Evaluation of Models’ Performance in the Test Set

We evaluated the models’ performance on the prospectively collected test cases and compared them with USFF. There was nearly perfect correlation and agreement between the linear multivariable model (r = 0.987, ICC = 0.977), the nonlinear multivariable model (r = 0.976, ICC = 0.975), and USFF. The correlation between the linear and nonlinear multivariable models (r = 0.956, ICC = 0.975) was also excellent but slightly lower.

**Figure 2:**
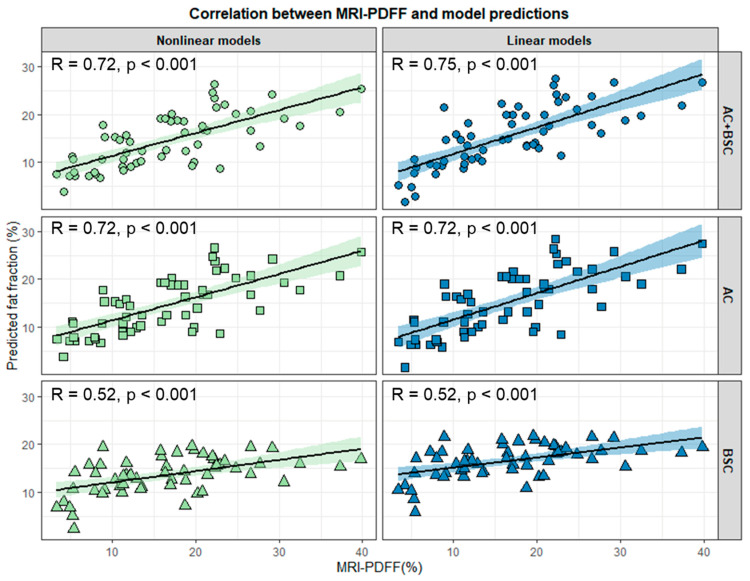
Correlation between predicted fat fraction and MRI-PDFF in the test set. Correlation plots were constructed between fat fraction predicted by different linear (blue markers) and nonlinear (green markers) models using the attenuation coefficient (AC), backscatter-distribution coefficient (BSC-D), or both as predictor variables and magnetic resonance fat fraction (MRI-PDFF). The plots show the correlation line (continuous line) and the 95% confidence intervals (shaded area). The labels in the upper left corner of the plots are the Pearson correlation coefficient (R) and its significance level (*p*).

The multivariable linear model produced the most accurate predictions (r = 0.752, *p* < 0.001) in the test set, which was in good agreement (ICC = 0.715) with MRI-PDFF (Figure 2). The accuracy and the agreement were both slightly lower with USFF (r = 0.722, ICC = 0.659), a nonlinear combined model (r = 0.719, ICC = 0.641), and the linear (r = 0.718, ICC = 0.688) and nonlinear (r = 0.718, ICC = 0.647) models based on AC only. The correlation was weak, and the agreement was poor with the TSI-based linear (r = 0.522, ICC = 0.367) and nonlinear (r = 0.52, ICC = 0.358) models (Supplementary Table S2).

The linear model combining AC and BSC-D (r = 0.602, *p* < 0.001, ICC = 0.529) and the USFF (r = 0.576, *p* < 0.002, ICC = 0.54) were the most reliable for prediction of low-grade (S0 and S1) steatosis. For cases with high-grade, S2, and S3 steatosis, the fit was weak with all models. The fit with the same multivariable linear model was only slightly better, and the agreement was weaker (r = 0.394, *p* < 0.029, ICC = 0.265) than that with a multivariable nonlinear model (r = 0.377, *p* < 0.037, ICC = 0.32) or a simple nonlinear AC model (r = 0.375, *p* < 0.037, ICC = 0.313). The USFF produced a worse and non-significant fit (r = 0.335, *p* < 0.066, ICC = 0.244) akin to the simple BSC-D models (linear: r = 0.155, *p* < 0.405, ICC = 0.055, *p* < 0.42; nonlinear: r = 0.152, ICC = 0.077) (Supplementary Table S3).

### 3.4. Grid Search of Ultrasound Parameters

**Figure 3:**
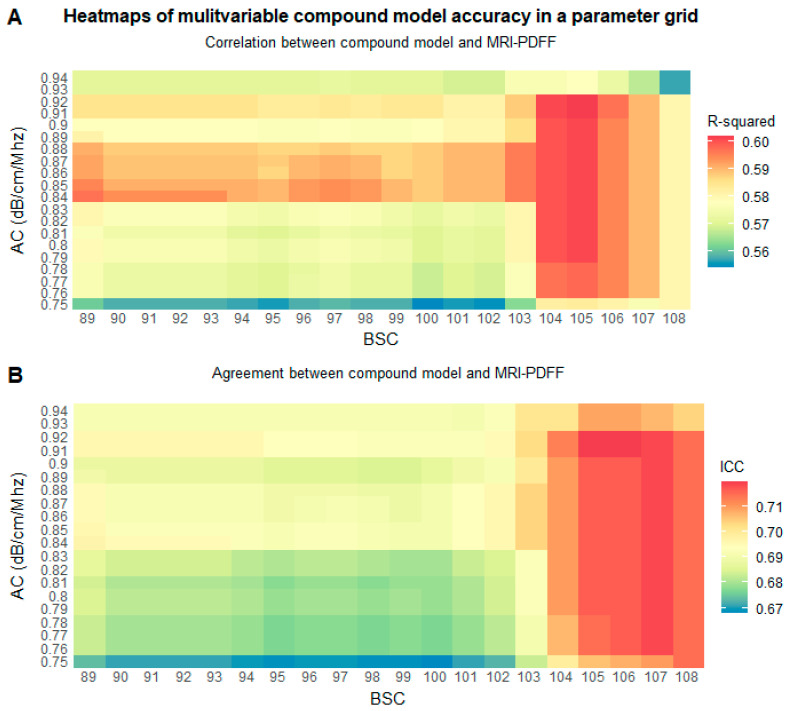
Heatmaps show the fit produced by a compound model in a parameter grid. We calculated (**A**) the R-squared and (**B**) the intraclass correlation coefficient (ICC) between predicted fat fraction and magnetic resonance fat fraction (MRI-PDFF) in the training set after switching to a nonlinear model from a multivariable linear model at various combinations of attenuation coefficient (AC) and backscatter-distribution coefficient (BSC-D) threshold values.

The detailed comparisons of the various fat fraction models indicated improving the accuracy of multivariable nonlinear and solely AC-based models and decreasing the accuracy of linear models in high-grade steatosis. Therefore, we performed a grid search of QUS parameters to identify the AC (between 0.75 and 0.94 dB/cm/MHz) and BSC-D (between 89 and 108) thresholds, above which switching from a linear to a nonlinear model could boost the overall accuracy. The grid search in the training set identified AC ≥ 0.84 dB/cm/MHz and BSC-D ≥105 as cut-off points for changing models (Figure 3).

### 3.5. Construction and Validation of Compound Models

**Figure 4:**
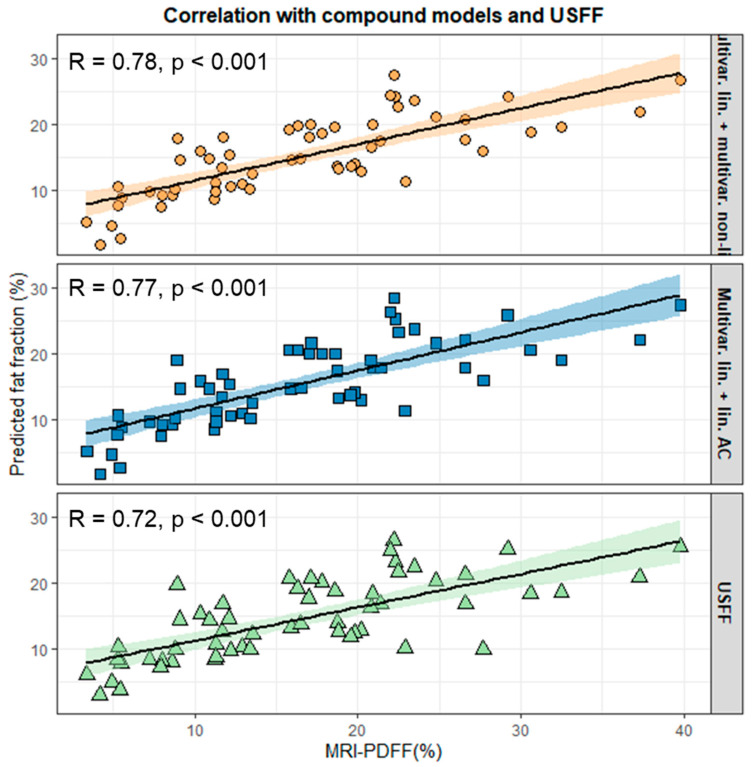
Correlation between predicted fat fraction and MRI-PDFF. The correlation plots show the fat fractions predicted by compound models and ultrasound fat fractions (USFFs, green triangles). The compound models combined the linear and nonlinear multivariable models (orange circle) or a linear multivariable model with a nonlinear AC model (blue rectangle). The correlation lines (continuous line) with 95% confidence intervals (shaded area) between MRI proton-density fat fraction (MRI-PDFF) and predicted fat fraction are also drawn. The labels in the upper left corner are the Pearson correlation coefficient (R) and the significance level (*p*).

We constructed two compound prediction models—one predicted fat fraction with a nonlinear multivariable model (CMT) and the other with a nonlinear AC model (CAC) above the grid-search-determined AC and BSC-D thresholds—and used the linear multivariable model for predicting cases with below-the-threshold parameter values. The fit of the CMT (LL = −202.55, *p* = 0.999) and the CAC (LL = −202.6, *p* = 1) models was not different from the linear multivariable model (LL = −202.55) in the training set. Meanwhile, CMT (r = 0.775, *p* < 0.001, ICC = 0.718) and CAC (r = 0.775, *p* < 0.001, ICC = 0.717) produced better correlation and agreement between the predicted fat fraction and MRI-PDFF than all other models, including the linear multivariable model and USFF in the test set (Figure 4).

**Figure 5:**
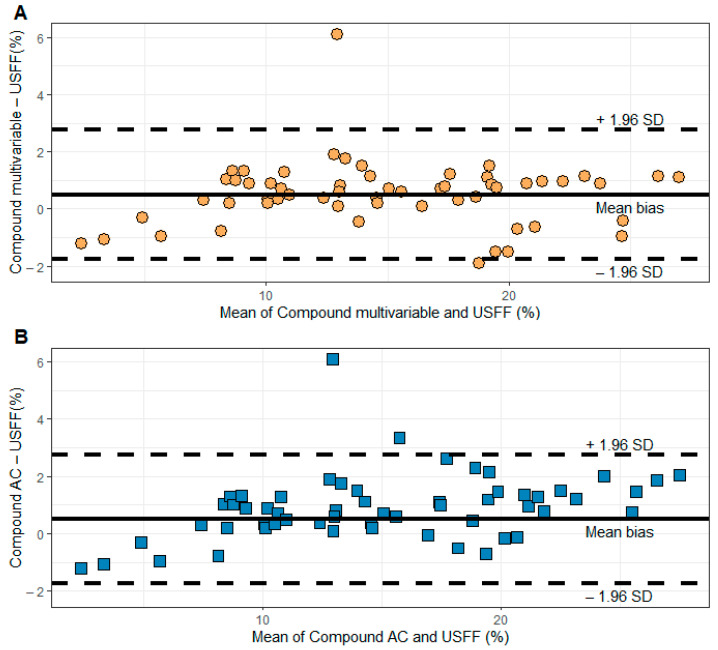
Bland–Altman analysis of the compound models and ultrasound fat fraction (USFF). The scatter plots display the difference and the mean of (**A**) the compound multivariable and (**B**) the compound AC models’ predictions and USFF. The mean bias (continuous line) and the upper and lower limits of agreement (dashed lines) are also shown.

The Blant–Altman analysis of the compound models and USFF revealed a mean difference of 0.5% and 0.9% and LOAs of −1.8–2.8% and −1.4–3.2% for CMC and CAC, respectively (Figure 5).

## 4. Discussion

In the present study, we conducted a side-by-side comparison of linear and nonlinear regression analyses to forecast hepatic fat fraction based on single or multiple QUS parameters. Our results show that with all models, the association between QUS-estimated fat fraction and the reference standard MRI-PDFF gets weaker at higher grades of steatosis. The linear models perform slightly better in S0 and S1 grades than nonlinear models. In contrast, the agreement produced by NLLS models was superior to linear models in S2 and S3 grades. Meanwhile, adding BSC-D to the linear model improved its accuracy in diagnosing low-grade steatosis. The models based on AC as the sole predictor variable resulted in better agreement than the multivariable models, including BSC-D in high-grade steatosis.

Previous studies agree that the ultrasound fat fraction is more accurate than conventional methods in detecting hepatic steatosis [24]. Meanwhile, the diagnostic accuracy of the QUS parameters is decreasing in high-grade steatosis. Accurate measurement of steatosis in all stages is clinically significant, as it has been demonstrated previously that high-grade steatosis detected with ultrasound was a predictor of MASH [25]. In addition, hepatic fat fraction is a quantitative biomarker that has an important role in monitoring the effects of diet and lifestyle modification in MASLD [26].

Currently, a limited number of publications are available on comparing the different QUS-based algorithms. The USFF was validated against MRI-PDFF in two studies, which found a good correlation (r = 0.76–0.82) in Asian patient populations. In our hands, the correlation for USFF was slightly lower (r = 0.72) in the test set [17,27]. Notably, the mean MRI-PDFF (mean = 16.5%) in our test set was higher than in the study, reporting a very strong correlation (mean = 7.9%), and comparable to the survey, reporting a strong correlation (mean = 16.45%). These findings support our observation that the accuracy of linear models is decreasing with increasing amounts of liver fat. The correlation between the USFF and a multivariable linear model was nearly perfect (r = 0.98). However, the predictions with the multivariable linear model trained on our patient population were slightly more than with USFF (r = 0.75 vs. 0.72, ICC = 0.72 vs. 0.66) in the test set. These findings indicate a slightly better fit of the in-house training model, which raises the possibility of significant differences between Asian and Caucasian populations that could influence the accuracy of the fat fraction predictions. Interestingly, we did not see improvement in the models’ fit when BMI or SLD was included as a third predictor variable. However, these findings may not be conclusive, as the patient population was almost uniformly overweight, and there was little variability in BMI and SLD.

Previous publications also evaluated nonlinear models, which reported strong and excellent (r = 0.82–0.91) correlations between MRI-PDFF and UDFF [16,28,29]. We found a consistently weaker correlation with a multivariable NLLS model both in the current test (r = 0.72) set and in a group of NAFLD patients (r = 0.74) published previously [20]. A study directly comparing UDFF and USFF found only a moderate correlation (r = 0.75) and good reliability (ICC = 0.84) [30]. One of the potential explanations for the difference is again the lower proportion of patients with high-grade steatosis (mean MRI-PDFF = 6.1–13.8%) in previous studies than in our test set. Another explanation for the lower inter-platform reproducibility of multiparametric fat fraction estimation is that BSC-D measured with TSI is not equivalent to the BSC measured for UDFF calculation. The authors of the prior study concluded that fat fraction values obtained by the two methods are not interchangeable due to the high inter-platform variability of the predictions [30]. Therefore, caution is necessary, as multivariable models may not be universally applicable to all platforms. Our results show that a compound model, which integrates the advantages of the linear and nonlinear methods, could enhance accuracy across all steatosis grades and may improve inter-platform reproducibility.

CAP is a highly popular, noninvasive method to quantify steatosis. It measures the attenuation of ultrasound waves emitted in a single beam in the liver. The correlation between CAP and MRI-PDFF was weaker compared to QUS-based methods in studies conducted on adult (r = 0.54) and pediatric (r = 0.55) populations [31,32]. In addition, no significant correlation was found when CAP was ≥331 dB/m [31]. One study compared CAP with UDFF and found good agreement (ICC = 0.86) [28]. We did not use CAP as a reference method in our research but developed univariable models with AC input. Meanwhile, a meta-analysis of multiple studies evaluating the diagnostic performance of AC for detecting steatosis found nearly equal specificity and sensitivity with different instruments [24]. Therefore, we assume that simple fat fraction models using AC as the only predictor variable could have good inter-platform reproducibility. The predictions of the univariable NLLS model (r = 0.72) closely matched with the multivariable (r = 0.72) model, reflecting the more significant influence of AC over BSC-D on the NLLS model outputs. Meanwhile, a univariable linear model (r = 0.72) was slightly less accurate than a multivariable one (r = 0.75), indicating that adding BSC-D improves the model’s fit.

After comprehensively evaluating the different models, we concluded that a multivariable linear model is more robust for predicting low-grade steatosis. This observation is in line with our previous report, where we found an excellent linear correlation (r = 0.9) if MRI-PDFF was <14.5% [20]. Meanwhile, NLLS models could be more accurate in high-grade steatosis. We completed a grid search of the AC and BSC-D parameters to test our hypothesis. Thresholds were set to all possible combinations of AC and BSC-D values to determine the optimal cut-off points, above which replacing the multivariable linear model with a multivariable NLLS or an AC-based NLLS model can improve overall accuracy and agreement. The optimal cut-off values were 0.84 dB/cm/MHz for AC and 105 for BSC-D in the training set. The compound models, which switched from a linear to a nonlinear model depending on the values of the QUS parameters, produced greater correlation (r = 0.77–0.78) and agreement (ICC = 0.72) than any other model. Recently, deep learning algorithms were devised to predict fat fraction using radiofrequency data or 2D images from ultrasound scans [18,19]. The advantage of a deep learning approach is that the number of tunable hyperparameters is much greater than in regression models, and an improved USFF accuracy is expected.

As TAI and TSI have been commercially available since 2020, only a handful of reports have been published on validating these ultrasound parameters [13,33,34,35]. Unfortunately, we could not find TAI and TSI data in any public repository that could be used as an outside validation set. Based on previous studies, which reported similarly good correlation with MRI-PDFF and similar diagnostic performance in detecting low- and high-grade steatosis to ours, it is a realistic expectation that the findings described in this paper can be reproduced during multi-center validation.

Our study has multiple limitations. First, patients were enrolled from a single center. Second, the cases in the training set were retrospectively selected. Third, training and test sets comprised a limited number of patients. Fourth, the test set was prospectively collected, but the clinical suspicion of MAFLD was high before the screening ultrasound. Thus, positive and high-grade steatosis cases were higher than in the general population. Fifth, the AC was measured with the TAI^TM^ and BSC-D with the TSI^TM^ application using the same scanner in all patients. Therefore, our findings cannot be generalized to QUS data obtained with scanners from other vendors.

## 5. Conclusions

The USFF and the multivariable linear regression model using AC and BSC-D predictor variables accurately diagnose low-grade steatosis. The nonlinear models using AC alone or together with BSC-D can be more reliable in high-grade steatosis. A compound model that switches from linear to nonlinear at ≥0.84 dB/cm/MHz AC and ≥105 BSC-D performs superior to other models.

## Figures and Tables

**Table 1:** Summary of demographic, imaging, and laboratory variables.

	Training Set (*n* = 60)	Test Set (*n* = 57)
AGE (years)	57.29 ± 13.64	50.33 ± 13.16
BMI (kg/cm^2^)	29.78 ± 4.18	30.57 ± 3.68
Female/male	26/34 (43.3%/56.6%)	26/31 (45.6%/54.4%)
T2DM	15 (25%)	7 (12.3%)
Ultrasound parameters		
SLD (cm)	2.16 ± 0.51	2.41 ± 0.47
PDFF (%)	14.12 ± 10.26	16.5 ± 8.47
USFF (%)	NA	14.58 ± 5.93
AC (dB/cm/MHz)	0.84 ± 0.14	0.85 ± 0.12
BSC-D	100.69 ± 9.22	99.61 ± 6.01
LS (kPa)	7.59 ± 3.4	5.38 ± 3.03
Blood tests		
ALT (U/L)	53 ± 41.66	64.75 ± 48.87
AST (U/L)	42 ± 35.26	44.33 ± 24.86
GGT (U/L)	105.07 ± 192.1	89.47 ± 99.87
TBIL (µmol/L)	15.37 ± 9.44	14.18 ± 8.31
ALB (g/L)	43.16 ± 3.94	46.06 ± 3.39
PLT (G/L)	232.86 ± 69.23	272.49 ± 55.21
INR	1.01 ± 0.08	1.07 ± 0.07
APRI	0.37 ± 0.27	0.35 ± 0.22
FIB4	1.56 ± 1	1.16 ± 0.72
NFS	−22.2 ± 11.86	−29.92 ± 2.74
HSI	41.5 ± 5.46	43.52 ± 6.71
Female/male	26/34 (43.3%/56.6%)	26/31 (45.6%/54.4%)
T2DM	15 (25%)	7 (12.3%)
Steatosis grade ^1^		
S0	16 (26.7%)	3 (5.3%)
S1	16 (26.7%)	23 (40.4%)
S2	12 (20%)	12 (21.1%)
S3	16 (26.7%)	19 (33.3%)
Fibrosis grade ^2^		
F0/1	7 (11.6%)	35 (61.4%)
F2	40 (66.6%)	17 (29.8%)
F3	9 (15%)	3 (5.3%)
F4	4 (6.6%)	2 (3.5%)

Categorical variables are presented as mean ± standard deviation, and continuous variables are presented as the number and percentage of the group. AC: attenuation coefficient; ALB: serum albumin; ALT: alanine aminotransferase; APRI: AST-to-platelet ratio index; AST: aspartate aminotransferase; BSC-D: backscatter-distribution coefficient; FIB4: fibrosis-4 index; GGT: gamma-glutamyl transferase; HSI: hepatic steatosis index; INR: international normalized ratio; *n*: number; LS: liver stiffness measured with 2D shear wave elastography; NA: not available; NFS: non-alcoholic fatty liver disease fibrosis score; PDFF: MRI proton-density fat fraction; PLT: platelet; SLD: skin-to-liver distance; T2DM: type 2 diabetes mellitus; ^1^ classification based on the PDFF measurement with cut-offs at 5%, 15%, and 20% [16,17]; ^2^ classification based on LS using the “rule of 4” with cut-offs at 5 kPa, 9 kPa, and 13 kPa [21,23].

**Table 2:** Results of multivariable and univariable regression analyses in the training set.

Regression Model	F-Statistics	b	R^2^	*p* Value ^#^
Multivariable	8.39	47.7 *	0.494 **	<0.001
AC	66.28	53.04	0.533	<0.001
BSC-D	20.96	0.573	0.265	<0.001
Age	0.27	−0.051	0.005	0.605
BMI	2.21	0.509	0.041	0.144
LS	0.003	−0.022	<0.001	0.955
SLD	5.86	5.99	0.092	0.019

AC: attenuation coefficient; b: beta coefficient; BMI: body mass index; BSC-D: backscatter-distribution coefficient; LS: liver stiffness; R^2^: R-squared; SLD: skin-to-liver distance; * only the beta of AC is shown; ** adjusted R-squared; ^#^ the level of significance was corrected with the Bonferroni method in case of the multiple univariate comparisons.

## Data Availability

The research data are available upon request.

## Supplementary Materials

The following supporting information can be downloaded at: https://www.mdpi.com/article/10.3390/diagnostics15020203/s1, Table S1: The parameters of the chemical-shift-encoded MRI sequence; Table S2: Correlation coefficients in the test set; Table S3: Intraclass correlation coefficients in the test set.

## Abbreviations

2D-SWE	Two-dimensional shear wave elastography
AC	Attenuation coefficient
ATI	Attenuation imaging
BMI	Body mass index
BSC	Backscatter coefficient
BSC-D	Backscatter-distribution coefficient
CAC	Compound nonlinear AC model
CAP	Controlled attenuation parameter
CMT	Compound nonlinear multivariable model
CSE-MRI	Chemical-shift-encoded magnetic resonance imaging
ICC	Intraclass correlation coefficient
IQR/M	Interquartile range median ratio
LL	Log-likelihood
LOAs	Limits of agreement
LR	Likelihood ratio
LS	Liver stiffness
MASH	Metabolic dysfunction-associated steatohepatitis
MASLD	Metabolic dysfunction-associated liver disease
MRI-PDFF	Magnetic resonance imaging proton-density fat fraction
MRS	Magnetic resonance spectroscopy
NLLS	Nonlinear least squares
OR	Odds ratio
QUS	Quantitative ultrasound
ROI	Region of interest
SLD	Skin-to-liver distance
SD	Standard deviation
T2DM	Type 2 diabetes
TAI	Tissue attenuation imaging
TSI	Tissue scatter-distribution imaging
UDFF	Ultrasound-derived fat fraction
UEFF	Ultrasound-estimated fat fraction
USFF	Ultrasound fat fraction

## References

[1] Wong V.W.-S., Ekstedt M., Wong G.L.-H., Hagström H. (2023). Changing Epidemiology, Global Trends and Implications for Outcomes of NAFLD. J. Hepatol..

[2] Rinella M.E., Lazarus J.V., Ratziu V., Francque S.M., Sanyal A.J., Kanwal F., Romero D., Abdelmalek M.F., Anstee Q.M., Arab J.P. (2023). A Multisociety Delphi Consensus Statement on New Fatty Liver Disease Nomenclature. J. Hepatol..

[3] Younossi Z.M., Kalligeros M., Henry L. (2024). Epidemiology of Metabolic Dysfunction-Associated Steatotic Liver Disease. Clin. Mol. Hepatol..

[4] Younossi Z.M., Stepanova M., Ong J., Trimble G., AlQahtani S., Younossi I., Ahmed A., Racila A., Henry L. (2021). Nonalcoholic Steatohepatitis Is the Most Rapidly Increasing Indication for Liver Transplantation in the United States. Clin. Gastroenterol. Hepatol..

[5] Abenavoli L., Spagnuolo R., Scarlata G.G.M., Scarpellini E., Boccuto L., Luzza F. (2023). Ultrasound Prevalence and Clinical Features of Nonalcoholic Fatty Liver Disease in Patients with Inflammatory Bowel Diseases: A Real-Life Cross-Sectional Study. Medicina.

[6] Horvath B., Allende D., Xie H., Guirguis J., Jeung J., Lapinski J., Patil D., McCullough A.J., Dasarathy S., Liu X. (2017). Interobserver Variability in Scoring Liver Biopsies with a Diagnosis of Alcoholic Hepatitis. Alcohol. Clin. Exp. Res..

[7] Nalbantoglu I.L.K., Brunt E.M. (2014). Role of Liver Biopsy in Nonalcoholic Fatty Liver Disease. World J. Gastroenterol..

[8] Segura-Azuara N.d.l.Á., Varela-Chinchilla C.D., Trinidad-Calderón P.A. (2022). MAFLD/NAFLD Biopsy-Free Scoring Systems for Hepatic Steatosis, NASH, and Fibrosis Diagnosis. Front. Med..

[9] Wan F., Pan F., Ayonrinde O.T., Adams L.A., Mori T.A., Beilin L.J., O’Sullivan T.A., Olynyk J.K., Oddy W.H. (2021). Validation of Fatty Liver Disease Scoring Systems for Ultrasound Diagnosed Non-Alcoholic Fatty Liver Disease in Adolescents. Dig. Liver Dis..

[10] Reeder S.B., Cruite I., Hamilton G., Sirlin C.B. (2011). Quantitative Assessment of Liver Fat with Magnetic Resonance Imaging and Spectroscopy. J. Magn. Reson. Imaging.

[11] Zsombor Z., Zsély B., Rónaszéki A.D., Stollmayer R., Budai B.K., Palotás L., Bérczi V., Kalina I., Maurovich Horvat P., Kaposi P.N. (2024). Comparison of Vendor-Independent Software Tools for Liver Proton Density Fat Fraction Estimation at 1.5 T. Diagnostics.

[12] Ferraioli G., Soares Monteiro L.B. (2019). Ultrasound-Based Techniques for the Diagnosis of Liver Steatosis. World J. Gastroenterol..

[13] Rónaszéki A.D., Budai B.K., Csongrády B., Stollmayer R., Hagymási K., Werling K., Fodor T., Folhoffer A., Kalina I., Győri G. (2022). Tissue Attenuation Imaging and Tissue Scatter Imaging for Quantitative Ultrasound Evaluation of Hepatic Steatosis. Medicine.

[14] Ferraioli G., Berzigotti A., Barr R.G., Choi B.I., Cui X.W., Dong Y., Gilja O.H., Lee J.Y., Lee D.H., Moriyasu F. (2021). Quantification of Liver Fat Content with Ultrasound: A WFUMB Position Paper. Ultrasound Med. Biol..

[15] Caussy C., Alquiraish M.H., Nguyen P., Hernandez C., Cepin S., Fortney L.E., Ajmera V., Bettencourt R., Collier S., Hooker J. (2018). Optimal Threshold of Controlled Attenuation Parameter with MRI-PDFF as the Gold Standard for the Detection of Hepatic Steatosis. Hepatology.

[16] Labyed Y., Milkowski A. (2020). Novel Method for Ultrasound-Derived Fat Fraction Using an Integrated Phantom. J. Ultrasound Med..

[17] Jeon S.K., Lee J.M., Cho S.J., Byun Y.-H., Jee J.H., Kang M. (2023). Development and Validation of Multivariable Quantitative Ultrasound for Diagnosing Hepatic Steatosis. Sci. Rep..

[18] Han A., Byra M., Heba E., Andre M.P., Erdman J.W., Loomba R., Sirlin C.B., O’Brien W.D. (2020). Noninvasive Diagnosis of Nonalcoholic Fatty Liver Disease and Quantification of Liver Fat with Radiofrequency Ultrasound Data Using One-Dimensional Convolutional Neural Networks. Radiology.

[19] Jeon S.K., Lee J.M., Joo I., Yoon J.H., Lee G. (2023). Two-Dimensional Convolutional Neural Network Using Quantitative US for Noninvasive Assessment of Hepatic Steatosis in NAFLD. Radiology.

[20] Kaposi P.N., Zsombor Z., Rónaszéki A.D., Budai B.K., Csongrády B., Stollmayer R., Kalina I., Győri G., Bérczi V., Werling K. (2023). The Calculation and Evaluation of an Ultrasound-Estimated Fat Fraction in Non-Alcoholic Fatty Liver Disease and Metabolic-Associated Fatty Liver Disease. Diagnostics.

[21] Barr R.G., Wilson S.R., Rubens D., Garcia-Tsao G., Ferraioli G. (2020). Update to the Society of Radiologists in Ultrasound Liver Elastography Consensus Statement. Radiology.

[22] Berglund J., Skorpil M. (2017). Multi-scale Graph-cut Algorithm for Efficient Water-fat Separation. Magn. Reson. Med..

[23] Barr R.G., Ferraioli G., Palmeri M.L., Goodman Z.D., Garcia-Tsao G., Rubin J., Garra B., Myers R.P., Wilson S.R., Rubens D. (2015). Elastography Assessment of Liver Fibrosis: Society of Radiologists in Ultrasound Consensus Conference Statement. Radiology.

[24] Ferraioli G., Barr R.G., Berzigotti A., Sporea I., Wong V.W.-S., Reiberger T., Karlas T., Thiele M., Cardoso A.C., Ayonrinde O.T. (2024). WFUMB Guidelines/Guidance on Liver Multiparametric Ultrasound. Part 2: Guidance on Liver Fat Quantification. Ultrasound Med. Biol..

[25] Nelson S.M., Hoskins J.D., Lisanti C., Chaudhuri J. (2020). Ultrasound Fatty Liver Indicator: A Simple Tool for Differentiating Steatosis From Nonalcoholic Steatohepatitis: Validity in the Average Obese Population. J. Ultrasound Med..

[26] Biciusca T., Stan S.I., Balteanu M.A., Cioboata R., Ghenea A.E., Danoiu S., Bumbea A.-M., Biciusca V. (2023). The Role of the Fatty Liver Index (FLI) in the Management of Non-Alcoholic Fatty Liver Disease: A Systematic Review. Diagnostics.

[27] Yin H., Fan Y., Yu J., Xiong B., Zhou B., Sun Y., Wang L., Zhu Y., Xu H. (2024). Quantitative US Fat Fraction for Noninvasive Assessment of Hepatic Steatosis in Suspected Metabolic-Associated Fatty Liver Disease. Insights Into Imaging.

[28] Wang P., Song D., Han J., Zhang J., Chen H., Gao R., Shen H., Li J. (2024). Comparing Three Ultrasound-Based Techniques for Diagnosing and Grading Hepatic Steatosis in Metabolic Dysfunction-Associated Steatotic Liver Disease. Acad. Radiol..

[29] Dillman J.R., Thapaliya S., Tkach J.A., Trout A.T. (2022). Quantification of Hepatic Steatosis by Ultrasound: Prospective Comparison With MRI Proton Density Fat Fraction as Reference Standard. Am. J. Roentgenol..

[30] Jeon S.K., Lee J.M. (2024). Inter-Platform Reproducibility of Ultrasound-Based Fat Fraction for Evaluating Hepatic Steatosis in Nonalcoholic Fatty Liver Disease. Insights Imaging.

[31] An Z., Liu Q., Zeng W., Wang Y., Zhang Q., Pei H., Xin X., Yang S., Lu F., Zhao Y. (2022). Relationship between Controlled Attenuated Parameter and Magnetic Resonance Imaging-Proton Density Fat Fraction for Evaluating Hepatic Steatosis in Patients with NAFLD. Hepatol. Commun..

[32] Shin J., Kim M.-J., Shin H.J., Yoon H., Kim S., Koh H., Lee M.-J. (2019). Quick Assessment with Controlled Attenuation Parameter for Hepatic Steatosis in Children Based on MRI-PDFF as the Gold Standard. BMC Pediatr..

[33] Zhu Y., Yin H., Zhou D., Zhao Q., Wang K., Fan Y., Chen K., Han H., Xu H. (2024). A Prospective Comparison of Three Ultrasound-Based Techniques in Quantitative Diagnosis of Hepatic Steatosis in NAFLD. Abdom. Radiol..

[34] Jeon S.K., Lee J.M., Joo I., Park S.J. (2021). Quantitative Ultrasound Radiofrequency Data Analysis for the Assessment of Hepatic Steatosis in Nonalcoholic Fatty Liver Disease Using Magnetic Resonance Imaging Proton Density Fat Fraction as the Reference Standard. Korean J. Radiol..

[35] Şendur H.N., Özdemir Kalkan D., Cerit M.N., Kalkan G., Şendur A.B., Özhan Oktar S. (2023). Hepatic Fat Quantification With Novel Ultrasound Based Techniques: A Diagnostic Performance Study Using Magnetic Resonance Imaging Proton Density Fat Fraction as Reference Standard. Can. Assoc. Radiol. J..

