# Supplementary material for: Construction of a Compound Model to Enhance the Accuracy of Hepatic Fat Fraction Estimation with Quantitative Ultrasound
Source: Diagnostics (Basel). 2025 Jan 17;15(2):203. doi: 10.3390/diagnostics15020203 (PMC11763894; doi:10.3390/diagnostics15020203)
Supplement: Supplementary file 1 [file diagnostics-15-00203-s001.zip › Table S1.pdf]

**Supplementary Table S1** The parameters of the chemical shift encoded MRI sequence

| **Acquisition parameter** | **Value** |
|---|---|
| Scanner | Philips Ingenia CX |
| Main magnetic field | 1.5 T |
| Sequence type | gradient echo |
| Sequence variant | steady state |
| Scan opiton | respiratory gated |
| Respiratory motion compensation | breath hold |
| Scan time | 3x20 s |
| Parallel reduction factor in plane | 1 |
| Number of averages | 1 |
| Acquistion type | 2D |
| Transmit coil type | Body |
| Recieve coil type | Multicoil |
| Repetition time | 150 ms |
| Echo time | 1.2 ms |
| Echo spacing | 1.2 ms |
| Echo train length | 12 |
| Flip angle | 10 degrees |
| Pixel bandwith | 2712 Hz |
| Acquistion matrix | 128*128 |
| Reconstruction diameter | 400 mm |
| Pixel spacing | 1.25/1.25 mm |
| Slice thickness | 5 mm |
| Interslice gap | 10 mm |
