# Supplementary material for: Construction of a Compound Model to Enhance the Accuracy of Hepatic Fat Fraction Estimation with Quantitative Ultrasound
Source: Diagnostics (Basel). 2025 Jan 17;15(2):203. doi: 10.3390/diagnostics15020203 (PMC11763894; doi:10.3390/diagnostics15020203)
Supplement: Supplementary file 1 [file diagnostics-15-00203-s001.zip › Table S2.pdf]

Supplementary table S2: Correlation coefficients in the test set

Dataset	Model name	Pearson r	CI low	CI up	R-squared	p-value
Test set	USFF	0.722	0.568	0.827	0.521	<0.001
	Linear AC+BSC	0.752	0.61	0.846	0.565	<0.001
	Linear AC	0.718	0.563	0.825	0.516	<0.001
	Linear BSC	0.522	0.302	0.688	0.272	<0.001
	Non-linear AC+ BSC	0.719	0.564	0.825	0.517	<0.001
	Non-linear AC	0.718	0.563	0.824	0.516	<0.001
	Non-linear BSC	0.520	0.3	0.687	0.27	<0.001
Test set, S0 & S1 grades	USFF	0.576	0.243	0.788	0.332	0.002
	Linear AC+BSC	0.602	0.28	0.802	0.363	0.001
	Linear AC	0.511	0.154	0.75	0.261	0.008
	Linear BSC	0.482	0.116	0.732	0.232	0.013
	Non-linear AC+ BSC	0.492	0.129	0.739	0.242	0.011
	Non-linear AC	0.494	0.132	0.74	0.244	0.01
	Non-linear BSC	0.468	0.099	0.724	0.219	0.016
Test set, S2 & S3 grades	USFF	0.335	-0.023	0.616	0.112	0.066
	Linear AC+BSC	0.394	0.045	0.656	0.155	0.029
	Linear AC	0.373	0.021	0.642	0.139	0.039
	Linear BSC	0.155	-0.211	0.483	0.024	0.405
	Non-linear AC+ BSC	0.377	0.025	0.645	0.142	0.037
	Non-linear AC	0.375	0.025	0.644	0.141	0.037
	Non-linear BSC	0.152	-0.216	0.479	0.023	0.42

AC: attenuation coefficient; BSC: backscatter-distribution coefficient; CI: confidence interval; USFF: ultrasound fat fraction; r: Pearson correlation coefficient; S0-S3 grades: steatosis grades were calculated from MRI-PDFF values with cut-off levels at 5%, 15%, and 20%.
