# Supplementary material for: Construction of a Compound Model to Enhance the Accuracy of Hepatic Fat Fraction Estimation with Quantitative Ultrasound
Source: Diagnostics (Basel). 2025 Jan 17;15(2):203. doi: 10.3390/diagnostics15020203 (PMC11763894; doi:10.3390/diagnostics15020203)
Supplement: Supplementary file 1 [file diagnostics-15-00203-s001.zip › Table S3.pdf]

Supplementary table S3 Intraclass correlation coefficients in the test set.

Dataset	Model name	ICC	CI low	CI up	p-value
Test set	USFF	0.659	0.474	0.786	<0.001
	Linear AC+BSC	0.715	0.561	0.821	<0.001
	Linear AC	0.688	0.523	0.803	<0.001
	Linear BSC	0.367	0.116	0.572	0.003
	Non-linear AC+ BSC	0.641	0.448	0.775	<0.001
	Non-linear AC	0.647	0.459	0.778	<0.001
	Non-linear BSC	0.358	0.114	0.562	0.002
Test set, S0 & S1 grades	USFF	0.54	0.212	0.761	0.001
	Linear AC+BSC	0.529	0.193	0.756	0.002
	Linear AC	0.461	0.116	0.712	0.005
	Linear BSC	0.193	-0.094	0.524	0.158
	Non-linear AC+ BSC	0.461	0.116	0.712	0.005
	Non-linear AC	0.46	0.114	0.711	0.005
	Non-linear BSC	0.371	-0.005	0.659	0.027
Test set, S2 & S3 grades	USFF	0.244	-0.064	0.528	0.063
	Linear AC+BSC	0.265	-0.059	0.552	0.058
	Linear AC	0.272	-0.053	0.557	0.053
	Linear BSC	0.055	-0.098	0.267	0.273
	Non-linear AC+ BSC	0.32	-0.004	0.593	0.027
	Non-linear AC	0.313	-0.01	0.587	0.029
	Non-linear BSC	0.077	-0.148	0.343	0.27

AC: attenuation coefficeint; BSC: backscatter-distribution coefficient; CI: confidence interval; ICC: interclass correlation coefficient (two-way mixed effect model, average values, absolute agreement); USFF: ultrasound fat fraction; S0-S3 grades: steatosis grades were calculated from MRI-PDFF values with cut-off levels at 5%, 15% and 20%.
